# Shionone relieves oxygen-glucose deprivation/reoxygenation induced SH-SY5Y cells injury by inhibiting the p38 MAPK/NF-κB pathway

**DOI:** 10.1186/s13019-024-02938-x

**Published:** 2024-07-12

**Authors:** Xiaoli Zhou, Xueting Luo

**Affiliations:** 1 Department of Neurology, The Affiliated Hospital of Hubei University of Chinese Medicine, Hubei Provincial Hospital of Traditional Chinese Medicine, Wuhan, 430061 China; 2 Department of Cardiovascular Medicine, The Affiliated Hospital of Hubei University of Chinese Medicine, Hubei Provincial Hospital of Traditional Chinese Medicine, No. 856 Luoyu Road, Hongshan District, Wuhan, 430061 China

**Keywords:** Shionone, Cerebral ischemia-reperfusion, OGD/R, p38 MAPK/NF-κB

## Abstract

**Background:**

Cerebral ischemia-reperfusion injury (I/R) can affect patient outcomes and can even be life-threatening. This study aimed to explore the role of Shionone in cerebral I/R and reveal its mechanism of action through the cerebral I/R in vitro model.

**Methods:**

SH-SY5Y cells were subjected to oxygen-glucose deprivation/reoxygenation (OGD/R) to induce cerebral I/R in vitro model. SH-SY5Y cells were treated with different concentrations of Shionone. Cell counting kit-8 and flow cytometry assays were used to detect cell viability and apoptosis levels. The levels of superoxide dismutase, catalase, and malondialdehyde were determined using their corresponding kits to examine the level of oxidative stress. The inflammation response was detected by IL-6, IL-1β, and TNF-α levels, using enzyme-linked-immunosorbent-assay. RT-qPCR was performed to measure the mRNA levels of p38 and NF-κB. Western blotting was used to quantify the apoptosis-related proteins and p38MAPK/NF-κB signaling pathway proteins.

**Results:**

Shionone exhibited no toxic effects on SH-SY5Y cells. Shionone inhibited OGD/R-induced cell apoptosis, improved the inflammatory response caused by OGD/R, and reduced the level of oxidative stress in cells. Western blot assay results showed that Shionone alleviated OGD/R-induced injury by inhibiting the activity of the p38 MAPK/NF-κB signaling pathway. The p38/MAPK agonist P79350 reversed the beneficial effects of Shionone.

**Conclusion:**

Shionone alleviates cerebral I/R and may thus be a novel therapeutic strategy for treating cerebral I/R.

## Introduction

Cerebral ischemia reperfusion injury (I/R) can often occur after ischemic stroke/vascular recanalization and cardiac arrest/cardiopulmonary resuscitation, seriously affecting the prognosis of patients. Oxidative stress is the main pathological factor behind cerebral I/R [1]. Brain tissue has a rich blood supply and contains high levels of polyunsaturated fatty acids. It has poor tolerance to ischemia and hypoxia—it is extremely vulnerable to oxidative stress attacks caused by ischemia and hypoxia, initiating a series of complex pathological processes in subsequent cascades [2, 3]. The recovery of neurological function in patients with cerebral I/R is difficult, often resulting in high mortality and disability rates, leading to a heavy economic burden on society and families [4]. Currently, as basic research in this field deepens our understanding of the pathological mechanisms of cerebral I/R, an expanding array of intervention targets and drugs with protective effects are being discovered [5]. However, discovering more unknown pathological mechanisms and understanding the interaction between pathological changes in various functional systems, as well as identifying effective drugs to find new targets for intervention, remain key challenges. It is imperative to address issues such as the transformation of basic research results into clinical applications.

Shionone is derived from the roots and rhizomes of a common Asteraceae plant, *Aster tataricus*. As per traditional medicine, it is warm in nature and bitter and spicy in taste and has the effect of eliminating phlegm and relieving cough [6]. Shionone is used to treat various diseases. Song et al. reported that Shionone can improve acute lung injury through the ECM1/STAT5/NF-κB pathway [7]. Shionone has also been reported to inhibit the progression of human breast cancer [8]. However, whether Shionone has a therapeutic effect on cerebral I/R and its potential mechanism of action remain unclear. Therefore, this study aimed to determine the effects of Shionone on cerebral I/R.

P38 mitogen-activated protein kinase/nuclear factor-kappa B (p38 MAPK/NF-κB) signaling pathway is a classical signaling pathway that is involved in several disease processes. Recently, it has been indicated that p38 MAPK/NF-κB is involved in LPS-induced acute lung injury [9]. Moreover, abnormal p38 MAPK/NF-κB pathway has been reported to result in Parkinson’s disease [10]. Li et al. showed that mulberry can improve diabetes through p38MAPK/NF-κB pathway [11]. Furthermore, a recent study revealed that p38MAPK/NF-κB pathway is involved in intestinal ischemia-reperfusion (I/R) injury [12]. However, it remains unknown whether Shionone can prevent cerebral I/R through the p38 MAPK/NF-κB pathway.

In summary, this study aimed to determine the effect of Shionone on cerebral I/R and illustrate its underlying mechanisms.

## Methods and materials

### Cell culture

Human neuroblastoma cells (SH-SY5Y) were purchased from the American Type Culture Collection (ATCC; Rockville, MD, USA). The cells were cultured in MEM/F12 (Biological Industries, Israel) containing 1% penicillin and streptomycin (Biological Industries, Israel) and 15% fetal bovine serum (Sigma-Aldrich, USA) in an atmosphere of 5% CO_2_ at 37 °C.

### Cell model

An in vitro cell model of cerebral I/R was established following previously described methods [13]. Briefly, cells were cultured in glucose-free medium under hypoxic conditions (1% O_2_ + 94% N_2_ + 5% CO_2_) at 37 °C for 4 h to establish an OGD model. The cells were then cultured in normal medium with normal oxygen content for 48 h for reperfusion.

After the model was established, the cells were treated with different concentrations of Shionone and P79350 (a p38/MAPK agonist; Selleck, China).

### Cell-counting Kit-8 (CCK-8)

Cell growth was examined using a CCK-8 kit (Fcmacs, Nanjing, China). SH-SY5Y cells were seeded in 96-well plates at 3 × 10^3^ cells/well and cultured with 10 µL solution for 1.5 h at 37 °C and 5% CO_2_ in the dark. The optical density (OD) values were calculated at 450 nm using an ultraviolet spectrophotometer (Infinite Pro, Tecan).

### LDH assay

The LDH levels in the cells were measured following co-culture with OGD/R or Shionone using LDH activity kits (Solarbio, Beijing). Following the manufacturer instructions, the OD value of each well was quantified at 490 nm to analyze LDH activity.

### Cell apoptosis

1 × 10^6^ SH-SY5Y cells were harvested in 500 µL solution comprising 5 µL Annexin V-FITC and 5 µL propidium iodide (Fcmacs, China) at room temperature in dark for 30 min. Subsequently, cell apoptotic rate was analyzed by flow cytometry (C6, BD, USA).

### Caspase-3 activity assay

Caspase-3 activity of the Shionone-treated cells was determined using a caspase-3 activity kit (Solarbio, China). Briefly, culture medium and cells digested with trypsin (Biological Industries, Israel) were harvested. Lysis buffer (Solarbio, China) was incubated with the supernatant for 15 min; the supernatant was obtained by centrifugation at 12,000 rpm for 15 min. The results were then analyzed using a microplate reader (Dragon Lab).

### Enzyme-linked immunosorbent assay (ELISA)

The culture medium was harvested after OGD/R and Shionone treatment and used for detecting the level of pro-inflammatory cytokines (IL-6, IL-1β, and TNF-α). The ELISA kit was obtained from R&D. All operations were performed according to the manufacturer’s protocol.

### Measurement of superoxide dismutase (SOD), catalase (CAT), and malondialdehyde (MDA)

Following OGD/R and Shionone treatment, the SOD, CAT, and MDA levels in the cells were measured using corresponding biochemical assay kits (Jiancheng, Nanjing). The OD value of each well was determined at 525 nm, following the manufacturer’s instructions.

### qPCR assay

Total RNA was isolated from the cells using ISOLATION TRIzol buffer^®^ (Multi Sciences, Hangzhou), following the supplier’s protocol, and cDNA was obtained by reverse transcribing RNA with a RT-PCR Kit (Yeasen, China). RT-qPCR was performed using PerfectStart^®^ Sybr qPCR Mix (Vazyme, Nanjing). The expression levels was calculated by the 2^−ΔΔCt^ assay.

### Western blot assay

OGD/R- and Shionone-treated cells were harvested using RIPA buffer (CST, USA) after lysis. A 10% gel was used to detach the proteins, which were then transferred to PVDF membranes (Whatman, USA). Next, 1 × PBST (Univ, China) and 5% non-fat milk powder (CST, USA) were used to block the membranes. The PVDF membranes were then incubated for 16 h with primary antibodies (Arigo, Taiwan, China), including antibodies against caspase-3 (ARG57512), p-p38 (ARG51850), p38 (ARG55258), p-p65 (ARG51518), p65 (ARG57479), and GAPDH. After blocking the membranes with secondary antibodies (Arigo, Taiwan, China) the following day, the pattern was presented using an image capture system (Wix, USA), and the grayscale value of the target was counted using ImageJ.

### Statistical analysis

Data are presented as the average ± standard deviation (SD) of three independent measurements. Unpaired Student’s t-test or one-way analysis of variance followed by Tukey’s post-hoc test was performed for data analysis. Statistical significance was set at *P* < 0.05.

## Results

### Shionone was non-toxic to SH-SY5Y

**Fig. 1:**
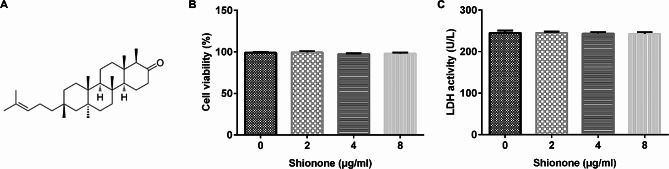
Effect of Shionone on hEECs. **(a)** The molecular structure of Shionone. **(b)** CCK-8 assay was performed to assess cell viability. **(c)** LDH detection kit was used to measure LDH activity. Data are presented as average ± SD of three independent experiments

CCK-8 and LDH analyses were performed to examine the SH-SY5Y cells treated with different concentrations of Shionone (0, 2, 4, and 8 µg/mL) for 24 h. Figure 1A shows the molecular structure of Shionone. The CCK-8 OD values indicated that Shionone did not exhibit toxicity to SH-SY5Y cells (Fig. 1B). Similarly, LDH activity indicated that Shionone had no effect on SH-SY5Y cells (Fig. 1C). These results confirmed that Shionone did not affect cell viability.

### Shionone inhibited OGD/R-induced cell viability reduction and apoptosis in SH-SY5Y cells

**Fig. 2:**
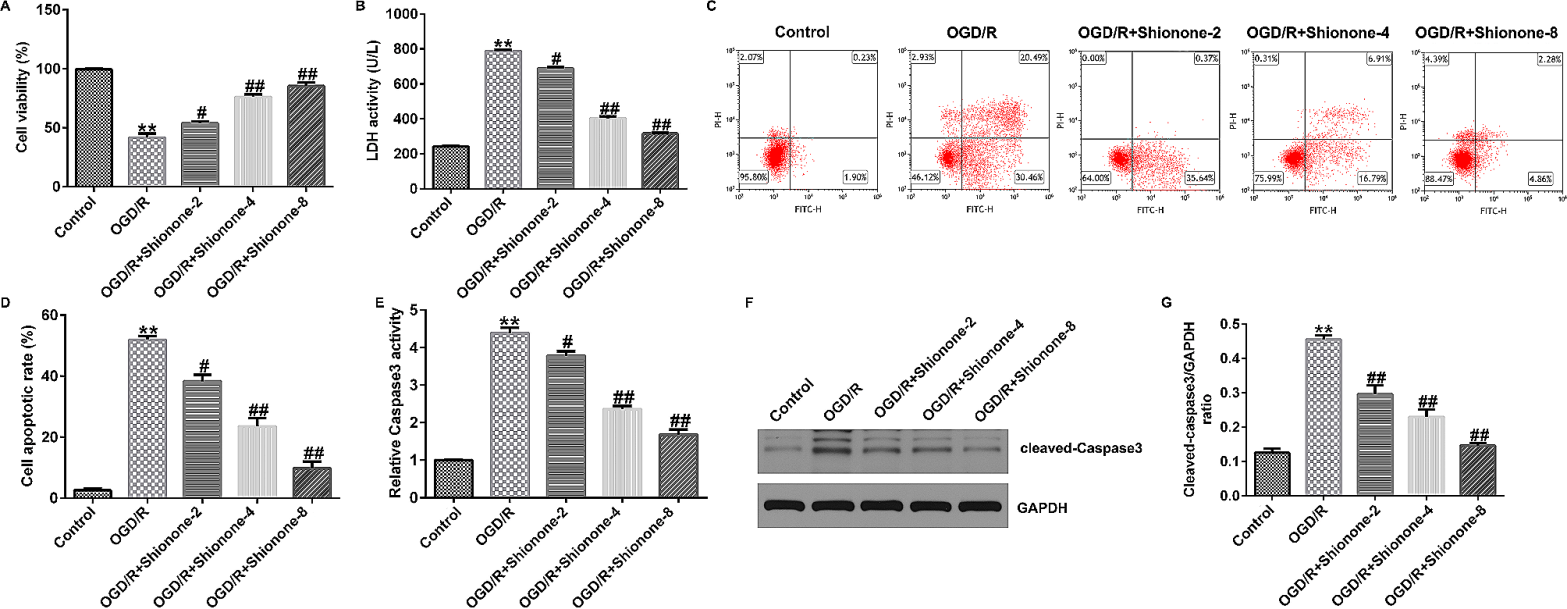
Effect of Shionone on apoptosis of SH-SY5Y cells. **a.** CCK-8 assay was performed to assess cell viability. **B.** LDH detection kit was used to measure LDH activity. **c–d.** FCM assay was conducted to assess cell apoptosis. **3.** Capase-3 activity detection kit was used to measure caspase-3 activity. **f–g.** Western blot was performed to quantify the patterns of apoptosis-related protein (cleaved-caspase-3). ** indicates *P* < 0.01 vs. control; # and ## indicates *P* < 0.05 and 0.01 vs. OGD/R, respectively. Data are presented as average ± SD of three independent experiments

To clarify the effect of Shionone on cerebral I/R, SH-SY5Y cells were treated with different concentrations (0, 2, 4, and 8 µg/mL) of Shionone for 24 h after OGD/R induction, followed by subsequent assays. The CCK-8 assay results showed that cell growth was repressed in the OGD/R group, but Shionone restored cell viability in a dose-dependent manner (Fig. 2A). the LDH level was clearly increased in the OGD/R group compared with the control group, but Shionone reversed this phenomenon (Fig. 2B). FCM results indicated that the level of apoptosis was increased in the OGD/R group, but Shionone could inhibit cell apoptosis in a dose-dependent manner (Fig. 2C and D). Meanwhile, caspase-3 activity was increased in the OGD/R group; however, Shionone repressed these effects (Fig. 2E). Additionally, western blotting results indicated that the level of cleaved-caspase-3 as well as the proportion of cleaved-caspase-3/GAPDH was elevated by OGD/R, and these effects were reversed by Shionone (Fig. 2F and G).

### Shionone suppressed inflammation and oxidative stress in OGD/R-induced SH-SY5Y cells

**Fig. 3:**
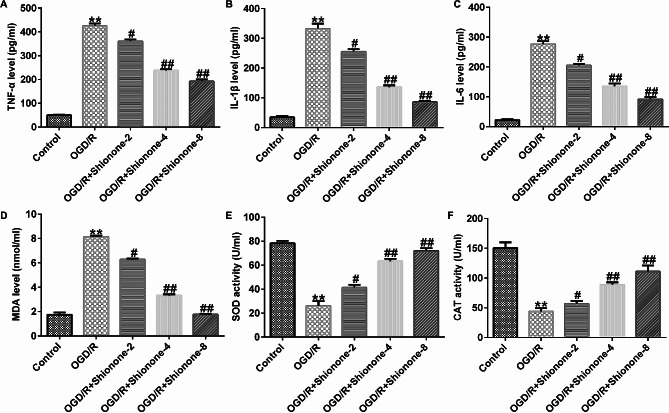
Impact of Shionone on inflammation and oxidative stress of OGD/R-induced cells. **a–c.** The concentrations of TNF-α, IL-1β, and IL-6 were detected using ELISA. **d–f.** The levels of MDA, SOD, and CAT. ** indicates *P* < 0.01 vs. control; # and ## indicates *P* < 0.05 and 0.01 vs. OGD/R, respectively. Data are presented as average ± SD of three independent experiments

To investigate the effects of Shionone on the inflammatory response and oxidative stress induced by OGD/R, the levels of TNF-α, IL-1β, and IL-6 were measured using ELISA. The levels of MDA, SOD, and CAT were determined using their corresponding kits. The levels of IL-6, TNF-α, and IL-1β were highly upregulated by OGD/R compared with the control; however, these effects were impaired by Shionone treatment (Fig. 3A and C). The MDA level was increased in the OGD/R group, and Shionone reversed this effect in a dose-dependent manner (Fig. 3D). Additionally, the activities of SOD and CAT were notably decreased in the OGD/R group but were rescued by Shionone (Fig. 3E and F). These results demonstrated that Shionone could suppress inflammation and oxidative stress in OGD/R-induced SH-SY5Y cells.

### Shionone inhibited OGD/R-induced p38 MAPK phosphorylation and NF-kB p65 phosphorylation

**Fig. 4:**
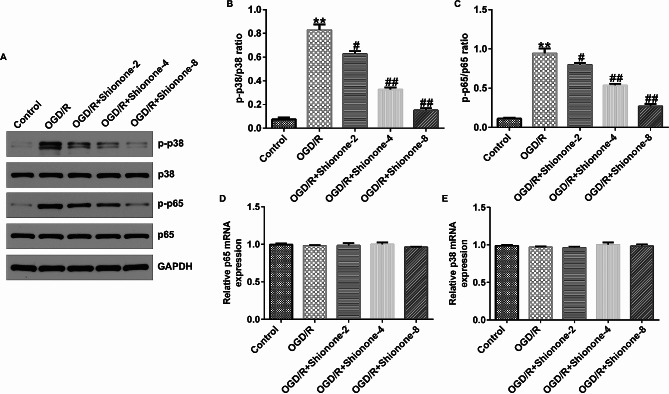
The level of p38 MAPK/NF-κB on Shionone-treated SH-SY5Y cells. **a–c.** The level and ratio of phospho-p38 MAPK and phospho-NF-kB were estimated using western blot. **d–e.** RT-qPCR was enacted to detect the level of p38 MAPK and NF-kB. ** indicates *P* < 0.01 vs. Control; # and ## indicates *P* < 0.05 and 0.01 vs. OGD/R, respectively. Data are presented as average ± SD of three independent experiments

Shionone attenuated OGD/R-induced cell damage. To explore the potential mechanisms of this phenomenon, we examined the p38 MAPK/NF-kB pathway using western blotting and qPCR. The expressions of p-p65 and p-p38 proteins and the ratios of p-p65/p65 and p-p38/p38 in SH-SY5Y cells were substantially increased in the OGD/R group compared with the control group, and this increase was inhibited by Shionone in a dose-dependent manner (Fig. 4A and C). Simultaneously, qPCR results showed that there was no significant difference in the mRNA expression of p38 and p65 among the groups (Fig. 4D and E).

### Shionone played a protective role in OGD/R-induced SH-SY5Y cell damage by inhibiting the p38 MAPK/NF-κB signaling pathway

**Fig. 5:**
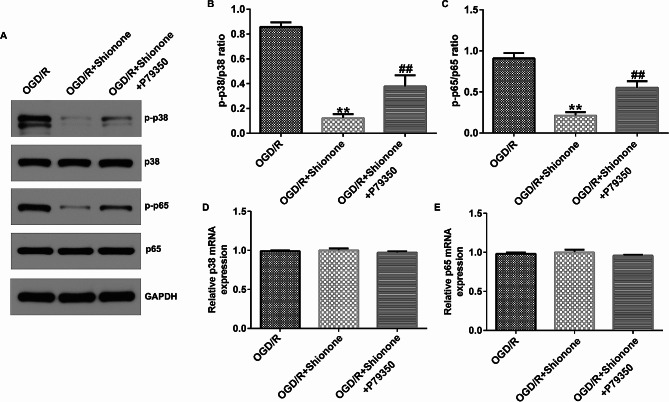
The effect of Shionone and P79350 on p38 MAPK/NF-κB pathway. **a–c.** The level and ratio of phospho-p38 MAPK and phospho-NF-kB were estimated using western blot. **d–e.** RT-qPCR was used to detect the level of p38 MAPK and NF-kB. ** indicates *P* < 0.01 vs. OGD/R; ## indicates *P* < 0.01 vs. OGD/R + Shionone. Data are presented as average ± SD of three independent experiments

To explore whether the protective effect of Shionone on cerebral I/R was mediated through the p38 MAPK/NF-kB signaling pathway, OCD/R-induced SH-SY5Y cells were treated with Shionone (8 µg/mL) and the p38/MAPK agonist P79350 (50 µM) for 24 h. Subsequent experiments were then performed. Compared with the OGD/R group, in the OGD/R + Shionone group, the protein expressions of p-p65 and p-p38 and the ratios of p-p65/p65 and p-p38/p38 in SH-SY5Y cells were notably disturbed (Fig. 5A and C). Compared with the OGD/R + Shionone group, P79350 significantly increased the protein expression of p-p65 and p-p38 and the ratios of p-p65/p65 and p-p38/p38 in SH-SY5Y cells. RT-qPCR revealed that the mRNA levels of NF-kB and p38 were not significantly different among the groups (Fig. 5D and E).

**Fig. 6:**
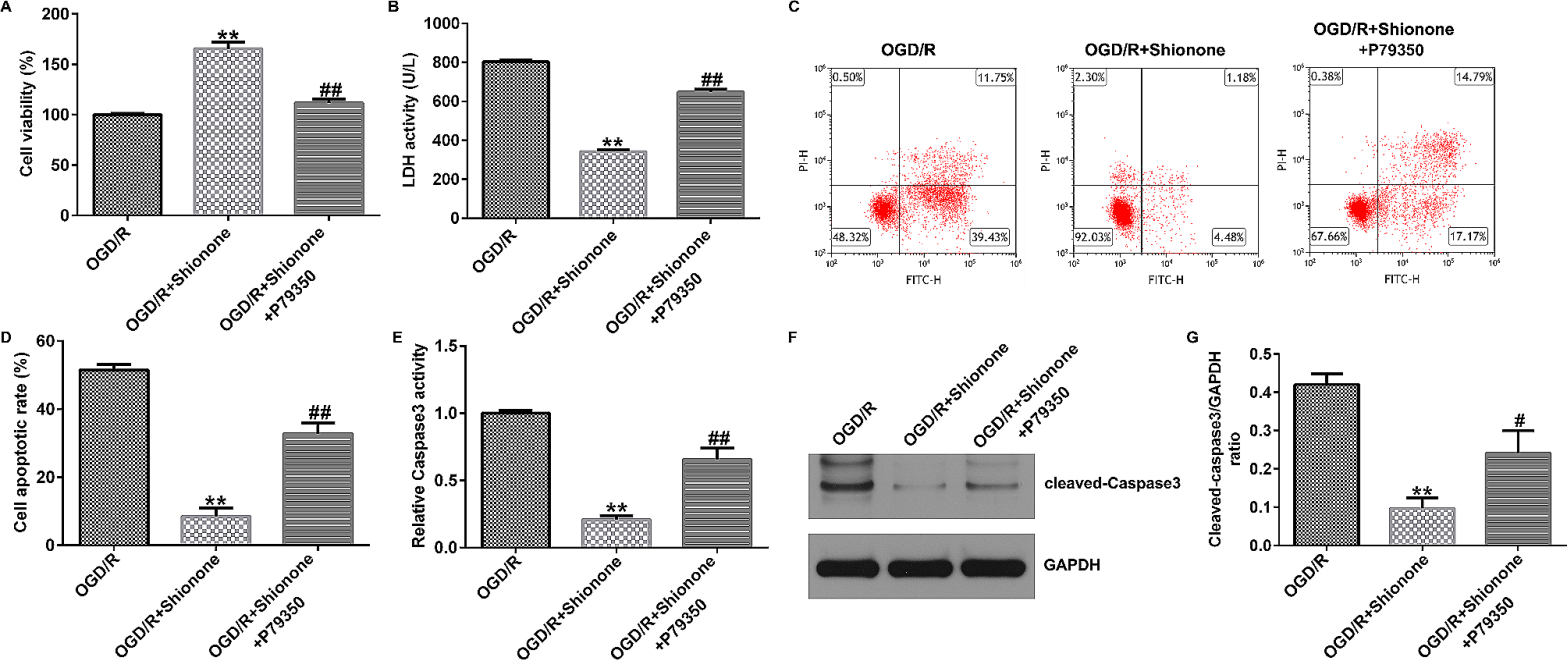
Shionone inhibited OGD/R-induced apoptosis in SH-SY5Y through p38 MAPK/NF-κB. **(a)** CCK-8 assay was performed to obtain cell viability. **(b)** LDH detection kit was used to measure LDH activity. **c–d.** Apoptosis rate in SH-SY5Y was confirmed by FCM assay. **e.** Caspase-3 activity detection kit was used to measure caspase-3 activity in SH-SY5Y. **f–g.** The pattern of cleaved-caspase-3 was verified by western blot. ** indicates *P* < 0.01 vs. OGD/R; ## indicates *P* < 0.01 vs. OGD/R + Shionone. Data are presented as average ± SD of three independent experiments

The viability of SH-SY5Y cells was significantly increased in the OGD/R + Shionone group, and P79350 significantly reduced the viability of SH-SY5Y cells (Fig. 6A). Compared with the OGD/R group, the OGD/R + Shionone group exhibited decreased LDH activity, but this phenomenon was reversed by P79350 (Fig. 6B). Shionone inhibited OGD/R-induced apoptosis, which was reversed by P79350 (Fig. 6C and D). The activity of caspase-3 was reduced in the OGD/R + Shionone group but increased in the P79350 co-culture (Fig. 6E). Additionally, the protein level of cleaved-caspase-3 and the proportion of cleaved-caspase-3 to caspase-3 were upregulated in OGD/R-induced cells, whereas Shionone substantially reduced the level of cleaved-caspase-3. However, co-culture with P79350 restored the ratio of cleaved-caspase-3/caspase-3 and the level of cleaved-caspase-3 (Fig. 6F and G).

**Fig. 7:**
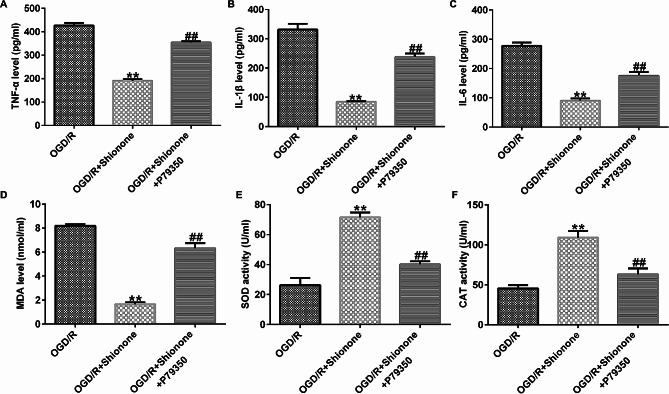
Shionone inhibited inflammation and oxidative stress induced by OGD/R in SH-SY5Y via p38 MAPK/NF-κB. **a–c.** The concentrations of TNF-α, IL-1β, and IL-6 were detected using ELISA. **d–e.** The levels of MDA, SOD, and CAT. ** indicates *P* < 0.01 vs. OGD/R; ## indicates *P* < 0.01 vs. OGD/R + Shionone. Data are presented as average ± SD of three independent experiments

The concentrations of TNF-α, IL-1β, and IL-6 were highly strengthened by OGD/R and impaired by Shionone treatment but increased again by P79350 (Fig. 7A and C). The release of MDA was significantly decreased, and the activities of SOD and CAT were significantly increased in the OGD/R + Shionone group compared with the OGD/R group. However, when the cells were co-treated with P79350, the MDA level was increased and the activities of SOD and CAT were decreased (Fig. 7D and F). These rescue experiments suggested that Shionone alleviates OGD/R-induced cell injury through the p38 MAPK/NF-kB signaling pathway.

## Discussion

As people’s living standards have improved and dietary habits have evolved, there is an increasing consumption of sugars and lipids. Owing to the limited absorption capacity of the body, blood viscosity can increase, and over time, it can easily lead to cardiovascular and cerebrovascular diseases, such as stroke [14]. At present, the primary focus in stroke treatment is to expediate the restoration of blood reperfusion; however, owing to the limited time window of thrombolysis, many patients cannot receive effective treatment in time [15]. Additionally, even if thrombolysis is used, brain tissue can be damaged after restoring blood reperfusion, which is called cerebral I/R [16]. Therefore, inhibiting cerebral I/R is an urgent issue that needs to be addressed. Consequently, it has garnered substantial attention in the present landscape of medical research.

Currently, foreign research on cerebral I/R has its limitations, mainly because of Western medicine components, such as antibiotics, that inhibit inflammatory factors. Some studies have shown that heme could effectively protect the blood-brain barrier, but these drugs have a single component and can only control the process of brain injury progression and cannot effectively reduce brain injury [17]. Moreover, Western medicine has side effects in clinical applications. Domestic research on brain injury is primarily focused on physical therapy and chemotherapy. Physical therapy mainly includes acupuncture and electrical stimulation, but their effects are often not obvious [18]. Chemotherapy mainly includes chemical drugs such as aspirin, β-blockers, angiotensin-converting enzyme inhibitors, and statins [19]. Therefore, it is particularly important to explore novel and safe therapies.

In our study, we found that Shionone could inhibit and attenuate OGD/R-induced cell damage, including cell apoptosis, inflammatory response, and oxidative stress, through the p38 MAPK/NF-kB pathway.

Shionone has various pharmacological activities such as anti-inflammatory and antioxidant properties. Recent studies have reported the biological activity of Shionone against several diseases. Zhang et al. showed that Shionone alleviates acute kidney injury by mediating macrophage polarization [20]. Du et al. indicated that Shionone targets pneumolysin to repress *Streptococcus pneumoniae* pathogenicity [21]. Yu et al. indicated that Shionone has anti-inflammatory activity [22]. Furthermore, a previous study demonstrated a potential treatment approach for COVID-19 using Shionone [23]. A recent study indicated that Shionone inhibits neuron apoptosis via the PI3K/Akt signaling pathway in spinal cord injury mice [24]. Various pathological processes are participated in the progression of cerebral I/R, such as oxidative stress, inflammatory response, and apoptosis [25–27]. Oxidative stress is caused by an imbalance between the antioxidant defense system and the rate of reactive oxygen species production, playing a crucial role in the process of cerebral I/R [26]. Cerebral I/R is closely related to inflammatory cytokines, which are regulatory factors in the mechanism of I/R induced inflammation [25]. Cerebral I/R can activate various cell death programs, such as apoptosis, necrosis, or autophagy related cell death. Among them, cell apoptosis is considered a key event in brain injury after cerebral ischemia [28]. Given this, we envision that Shionone may participate in cerebral ischemia-reperfusion injury by regulating the inflammatory response, oxidative stress, and apoptosis. However, no study has investigated the effect of Shionone on cerebral I/R. Therefore, we explored the effects of Shionone on cerebral I/R in the present study. The findings of this study indicated that Shionone inhibited OGD/R-induced SH-SY5Y cell apoptosis, improved the inflammatory response caused by OGD/R, and reduced the level of oxidative stress in cells.

p38 MAPK/NF-κB signaling is a critical cytoprotective mechanism. There is growing evidence suggesting that the p38 MAPK/NF-κB signal pathway plays a critical function in the development of human diseases [29–32]. Previous studies have shown that cerebral ischemia is closely related to p38MAPK [33]. Due to the involvement of p38MAPK activation in ischemia-induced neural injury, blocking it can protect brain tissue from ischemic damage by reducing the production of inflammatory mediators and blocking the inflammatory process [33, 34]. As a nuclear target of the MAPKs pathway, NF-κB plays a crucial role in the inflammatory response process and is activated in cerebral I/R [35]. In our research, we found that Shionone improved OGD/R-induced cell damage via the p38 MAPK/NF-κB pathway.

However, there were some limitations of this study. We only studied the effect of Shionone treatment on OGD/R-induced SH-SY5Y cells for 24 h, and establishing different time points (such as 12 h, 24 h, 48 h, 72 h) to study the effects of Shionone on OGD/R induced SH-SY5Y cells injury will make our research more convincing. Besides, this study did not establish an OGD/R + P79350 treatment group, which will make the research conclusions more credible. Moreover, it is not clear whether Shionone can pass through the blood-brain barrier and whether it has an effect on the real animal model of cerebral infarction or patients, and this study only conducted in vitro studies without in vivo experiments. We will perform these issues in the future.

In conclusion, this study demonstrated that Shionone can mitigate OGD/R-induced cerebral I/R in vitro and that Shionone can decrease inflammation and repress p38 MAPK/NF-κB activation in OGD/R-induced SH-SY5Y cells. Therefore, this study indicates Shionone may be a potential therapeutic agent for treating cerebral I/R.

## Data Availability

The datasets used and/or analyzed during the current study are available from the corresponding author upon reasonable request.

## Abbreviations

CAT: Catalase
CCK-8: Cell-counting kit-8
Cerebral I/R: Cerebral ischemia reperfusion injury
ELISA: Enzyme-linked immunosorbent assay
I/R: Ischemia-reperfusion
MAPK: Mitogen-activated protein kinase
MDA: Malondialdehyde
NF-κB: Nuclear factor-kappa B
OD: Optical density
SOD: Superoxide dismutase
SD: Standard deviation
